# Management of Massively Enlarged Multiple Hydatid Cysts in the Liver of a Pediatric Patient

**DOI:** 10.7759/cureus.1643

**Published:** 2017-09-02

**Authors:** Faizan Yasin, Haider Ghazanfar, Salman Assad, Sumbal A Bhatti

**Affiliations:** 1 Neurology, State University of New York at Buffalo; 2 Internal Medicine, Newark Beth Israel Medical Center; 3 Department of Medicine, Shifa International Hospital, Islamabad, Pakistan; 4 Pediatrics, Ittefaq Hospital (Trust)

**Keywords:** hydatid disease, liver cyst, echinococcus

## Abstract

Multiple hydatid cysts in the liver are a very rare occurrence in childhood. We present a similar case in a nine-year-old girl, a resident in a rural community, who presented with two hydatid cysts in her liver. The cysts were operated upon by a pediatric surgeon. The laboratory findings of this patient showed peripheral blood eosinophilia, elevated white blood cells, and liver enzymes. The serology was positive. The ultrasonography showed cystic masses in the liver, and the diagnosis of hydatid cysts was eventually confirmed by computed tomography (CT) of the abdomen.

## Introduction

Hydatid disease is a parasitic infestation caused by Echinococcus spp. Mainly, cattle and sheep are the intermediate hosts, and dogs are the definitive hosts. Accidental ingestion of soil, food, or water that is contaminated by fecal matter from an infected dog is the most common mode of its transmission. It causes significant morbidity and mortality. In the majority of cases, the most frequently infected organ is the liver (75%), followed by the lungs (15%) [1]. It is considered to be one of the most frequent causes of liver masses. Other organs can also be affected by this disease, including the brain, heart, spleen, breast, and bone [2]. The diagnostic modality consists of abdominal ultrasound and computed tomography (CT) scan. The management of the disease comprises various techniques including medical treatment, aspiration of the cyst, and its surgical removal [3]. We report a similar case of multiple, massively enlarged hydatid cysts in the liver that was managed successfully in this hospital.

## Case presentation

A nine-year-old female, a resident of Afghanistan, presented to the Medical Outpatient clinic at Ittefaq hospital in Lahore with the complaint of pain in the abdomen for two months and decreased appetite, bouts of vomiting, and constipation for one month. The patient was in her usual state of health two months back when she gradually developed pain in the right hypochondrium, which was intermittent, mild to moderate in intensity, non-radiating, with no aggravating or relieving factors. The patient had frequent episodes of vomiting for one month, mostly one to two episodes per day and moderate in amount; it contained food particles and was non-bloody, nonbilious, and non-projectile. The patient denied any history of fever, jaundice, or a cough.

The past medical history was not significant. The patient was delivered in Afghanistan via spontaneous full-term vaginal delivery. There were no postnatal complications. There was no history of hospital admission at birth. She was breastfed for one year, then started on cow’s milk, and weaning was started at six months of age. She was fully vaccinated and achieved all developmental milestones appropriate for her age. The patient has two siblings who are healthy. Further history revealed that they have a pet cat at home.

On general physical examination, the patient was lying comfortably on the bed with a temperature of 98°F, respiratory rate of 24/min, heart rate of 80/min, blood pressure of 90/60 mmHg, with pallor, no clubbing, cyanosis, lymphadenopathy or any visible rashes on the skin. The abdomen was soft, non-distended, non-tender, the liver was palpable (3 cm below right costal margin), the spleen was not palpable, and bowel sounds were audible. Bilateral equal air entry in lungs was observed on auscultation. S1 + S2 heart sounds were present, and no murmurs were observed. The Glasgow coma scale (GCS) was 15/15. Muscle tone, power, and reflexes were normal in both upper and lower limbs. Based on the history and physical examination, a differential diagnosis of hepatocellular carcinoma, hepatic cysts, and the hepatic abscess was formulated.

**Figure 1:**
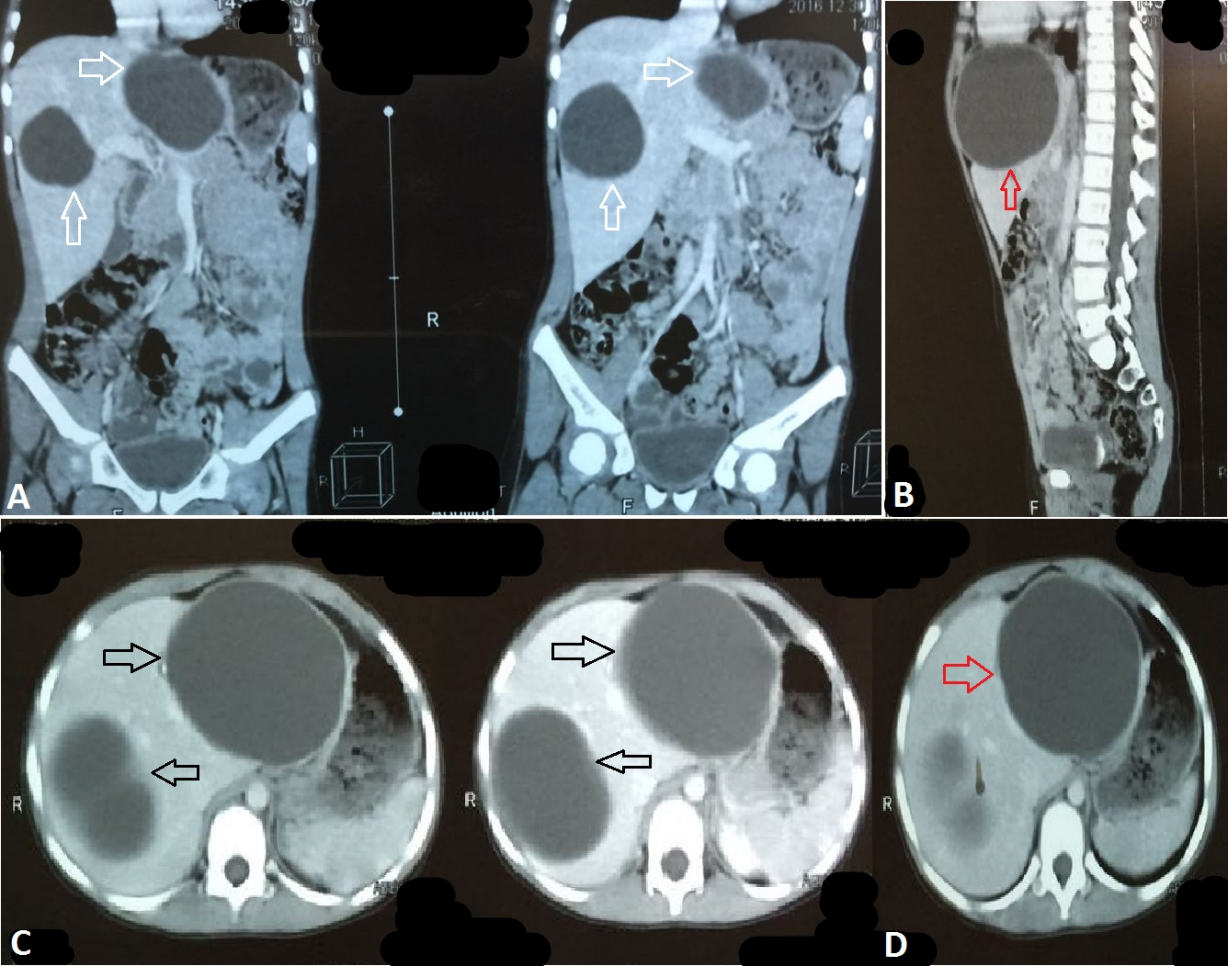
Computed Tomography Scan Findings Computed tomography (CT) scan showing two enlarged cysts in frontal view (white arrows in A). Massive cyst (red arrows in B and D). Two enlarged cysts in cross sectional view (black arrows in C).

A number of investigations were ordered including complete blood cell count (CBC), liver function tests (LFT), renal function tests (RFT), blood grouping and cross-match, viral markers, urine routine examination (R/E), erythrocyte sedimentation rate (ESR), ultrasonography (USG) of abdomen, abdominal CT scans, thyroid profile, immunoglobin E levels (IgE), and vitamin D levels. Surgical consultation was also advised. The laboratory investigation results are shown in Table 1 and Table 2. Serum IgE levels were raised to 1685 UI/ml (normal value is less than 90 UI/ml). USG abdomen findings showed an enlarged liver measuring 17 cm, normal parenchyma and smooth surface, portal vein of normal caliber, and multiple cysts with the largest cysts seen in the left lobe (88 × 83 mm) as well as in the right lobe (71 × 67 mm). No solid element was noted. Normal gallbladder, common bile duct, intrahepatic ducts, with only minimal ascites were seen. Multiple lesions in the liver, some having internal echoes, and some with more echogenic internal components consistent with hydatid cyst disease were found. CT scans of the abdomen showed multiple hepatic cysts, with few having neural calcification (Figure 1). On the basis of these investigational findings, a final diagnosis of hydatid cyst disease of the liver was made.

**Table 1:** Complete Blood Count with Differential Complete blood count (CBC); WBC: white blood cell; RBC: red blood cell; HCT: hematocrit; MCV: mean corpuscular volume.

Test(s)	Result	Normal Value	Unit
BLOOD COMPLETE PICTURE			
WBC Count	8.52	4 - 11	10^3/uL
RBC Count	4.67	4 - 5.2	10^6/uL
Hemoglobin	12.7	10.8 - 15.6	g/dL
HCT	37	33 - 45	%
MCV	79.2	79 - 95	fL
Platelets	269	150 - 450	10^3/uL
DIFFERENTIAL COUNT			
Neutrophils	36	34 - 70	%
Lymphocytes	46	19 - 52	%
Eosinophils	8	1 - 6	%

**Table 2:** Further Laboratory Investigations

Tests	Results (day of admission)	Results (1 week from day of admission)	Normal Values
Total bilirubin	0.3	0.6	0.1 - 1 mg/dL
Alanine aminotransferase (ALT)	28	33	5 - 55 U/L
Aspartate aminotransferase (AST)	34	34	5 - 38 U/L
Alkaline phosphatase (ALP)	66	65	35 - 290 U/L
Serum total protein	6.8	6.4	6.8 g/L
Serum albumin	2.7	3.2	3.8 - 5.4 g/L
Gamma glutamyl transferase (GGT)	44	49	9 - 48 U/L

**Figure 2:**
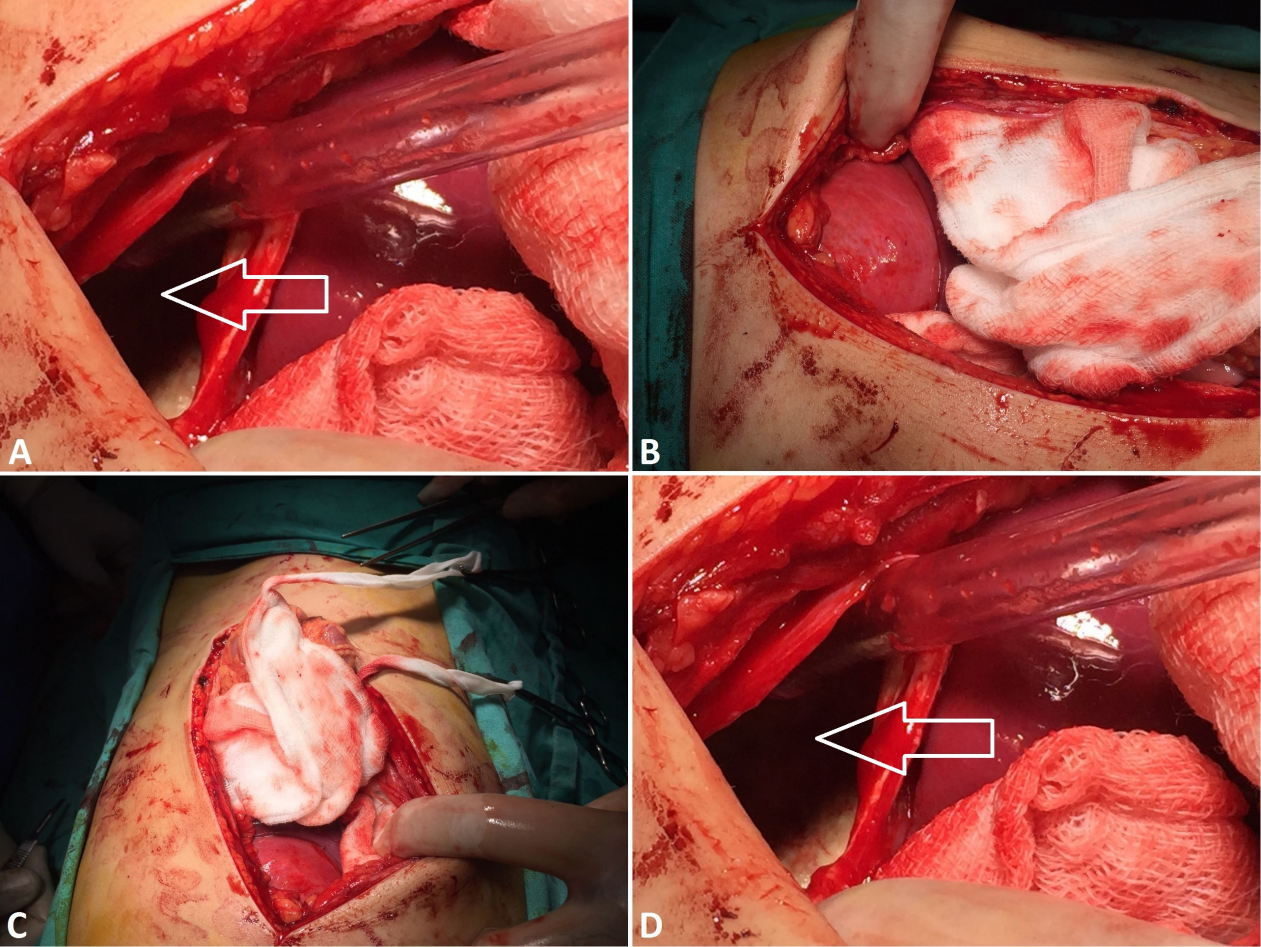
Surgical Findings Hydatid cysts (white arrows in A and D) aspirated and refilled with H_2_O_2_, then re-aspirated and cysts removed.

The patient was admitted, symptomatic treatment was started, and levamisole was given. The pediatric surgeon was also involved. The surgery was planned. Under aseptic measures, a midline incision was made, the abdominal wall was opened, and the cavity was examined. The hydatid cysts were aspirated, refilled with hydrogen peroxide (H_2_O_2_) then re-aspirated, and the cysts were removed. The abdominal cavity was washed with Pyodine and normal saline. A drain was placed, the abdominal wall was closed, and skin sutures were applied (Figure 2). The findings included two hydatid cysts from which 270 cc (left lobe) and 180 cc (right lobe) of fluid were aspirated.

On discharge, the patient was prescribed Zentel 200 mg orally, two tablets daily for 28 days. Repeat LFTs after 28 days were also advised.

## Discussion

A hydatid cyst is considered to be one of the major medical problems in underdeveloped countries, and proper measures must be taken to overcome this problem. The accidental ingestion of Echinococcus spp. eggs by people in close contact with dogs can lead to this disease. Patients are usually asymptomatic. However, they can be diagnosed when they present with nonspecific signs and symptoms and also on routine ultrasound and lab findings. Hydatid cysts usually involve the liver and lungs. Abdominal ultrasounds help in diagnosing this condition, and CT scans help in confirming the lesions in the liver. In this case, surgery was performed on the patient to remove the hydatid cyst. This also helps in preventing the recurrence of the cyst as well as its complications, such as infection, cholangitis, obstructive jaundice, and anaphylactic shock [4]. The disease can be prevented if the transmission cycle is broken [5]. Public awareness and maintaining proper hygiene will help control the spread of this disease.

## Conclusions

Massively enlarged hydatid cysts in the liver pose a significant risk of rupture, especially if located superficially. Ensuring proper hygiene is essential in preventing the spread and transmission of the parasite Echinococcus spp. Efforts must be made to ensure proper sanitation to prevent the occurrence of such pathologies, especially in underdeveloped countries and rural areas.
